# eRP arrangement: a strategy for assembled genomic contig rearrangement based on replication profiling in bacteria

**DOI:** 10.1186/s12864-017-4162-z

**Published:** 2017-10-13

**Authors:** Nobuaki Kono, Masaru Tomita, Kazuharu Arakawa

**Affiliations:** Institute for Advanced Biosciences, Keio University, Mizukami 246-2, Kakuganji, Tsuruoka, Yamagata 997-0052 Japan

**Keywords:** Bacterial genome, De novo sequencing, Experimental replication profiling, Assemble

## Abstract

**Background:**

The reduced cost of sequencing has made de novo sequencing and the assembly of draft microbial genomes feasible in any ordinary biology lab. However, the process of finishing and completing the genome remains labor-intensive and computationally challenging in some cases, such as in the study of complete genome sequences, genomic rearrangements, long-range syntenic relationships, and structural variations.

**Methods:**

Here, we show a contig reordering strategy based on experimental replication profiling (eRP) to recapitulate the bacterial genome structure within draft genomes. During the exponential growth phase, the majority of bacteria show a global genomic copy number gradient that is enriched near the replication origin and gradually declines toward the terminus. Therefore, if genome sequencing is performed with appropriate timing, the short-read coverage reflects this copy number gradient, providing information about the contig positions relative to the replication origin and terminus.

**Results:**

We therefore investigated the appropriate timing for genomic DNA sampling and developed an algorithm for the reordering of the contigs based on eRP. As a result, this strategy successfully recapitulates the genomic structure of various structural mutants with draft genome sequencing.

**Conclusions:**

Our strategy was successful for contig rearrangement with intracellular DNA replication behavior mechanisms and can be applied to almost all bacteria because the DNA replication system is highly conserved. Therefore, eRP makes it possible to understand genomic structural information and long-range syntenic relationships using a draft genome that is based on short reads.

**Electronic supplementary material:**

The online version of this article (10.1186/s12864-017-4162-z) contains supplementary material, which is available to authorized users.

## Background

In microbiology, whole genome sequencing is no longer a unique type of analysis, and it is now performed within individual research studies [1, 2]. This recent change is due to improvements in massively parallel sequencing technologies with dramatically reduced costs [3], as well as improvements in bioinformatics software for efficiently processing large amounts of data [4, 5]. In particular, characteristic assemblers for various situations have been developed, such as the long-read assemblers Canu [6] or HINGE [7], as well as scaffolding tools [8]. Furthermore, SPAdes is known as an assembler for single-cell sequencing data [9]. It has been used for bacterial genome assembly [10]. These assemblers have been compared and examined under various conditions, and each has been successful for the appropriate genome projects [10–13]. However, even with these assemblers, it is not easy to obtain information about the genomic structure.

The bacterial genome structure can be observed in various layers, such as the base compositional bias, gene strand bias, and oligomer skew [14–19]. To address the energy efficiencies [20, 21] and environmental pressures [22, 23], these genomic structures have been established due to complex interactions among biological, chemical, and physical mutagens over long evolutionary timescales [24, 25]. Specifically, bacterial genome sequences show different types of base compositions that cannot be explained by phylogenetic classifications [26, 27]. Additionally, the genomic structure is not only a crucial evolutionary trajectory, but it also plays a central role in biological processes [28–30]. Most existing assemblers set up contigs mathematically using numerous sequence reads, and each contig is assembled individually. Therefore, there is no information to interrelate the genome position of each assembled contig, and an analysis of the genomic structure requires the finished genome. This problem is not limited to de novo sequencing. Even if there is a closely related reference species, this problem cannot be solved by simple alignment comparisons because there may be large-scale mutations that affect the genome structure, including inversions, insertions or deletions. Hence, it is necessary to use both mathematical and biological information.

**Fig. 1:**
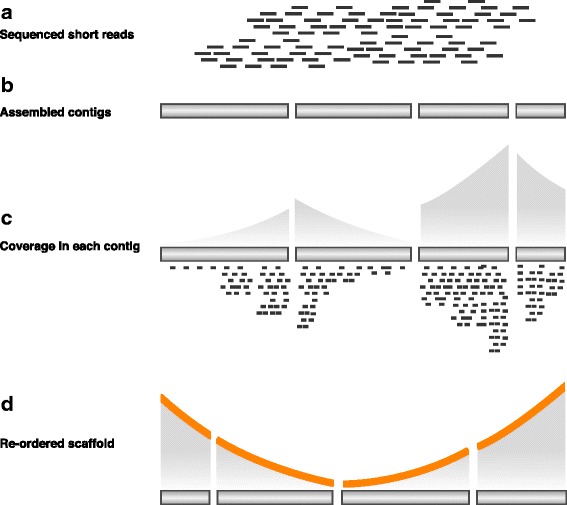
The eRP procedure consists of the following four steps: **a** genomic DNA extraction at an appropriate growth phase, **b** sequencing and short-read assembly, **c** coverage calculation at each contig by read mapping, and **d** the arrangement of the order and orientation of the contigs in a continuous coverage gradient

In this paper, we introduce the experimental replication profiling (eRP) arrangement strategy, which analogizes the positional relationship between contigs based on the biological molecular behavior information. eRP is a technique for calculating the differences in the DNA copy number among each genome position according to sequence read coverage during the exponential phase [31]. This technique is widely applicable not only to *Escherichia coli* and *Bacillus subtilis* but also to other bacterial genomes obtained by metagenome analysis [32]. Furthermore, the gradient tendency in the DNA copy number is based not on the genome sequence information but on the genomic structure-dependent replication behavior [28]. Hence, the concept of the eRP arrangement system is to assemble the contigs using genome sequencing data that are sampled during the exponential phase, calculate the DNA copy number gradient in each contig, and rearrange the order and orientation based on the gradient shape (Fig. 1).

## Results

### Relationship between the growth phase and DNA copy number gradient

The primary strategy of eRP arrangement is the use of replication behavior as a biological guideline for arranging the contigs (Fig. 1). At this time, the replication behavior is monitored as a DNA copy number coverage gradient over the entire genome via the eRP method [28, 31, 33], and its clarity depends on the sampling timing. Previous studies have found that the optimal sampling time is during the exponential phase [28]. To determine the growth range limit at which a clear coverage gradient appears, we performed eRP using *B. subtilis* WT genomic DNA at each time point according to the OD_600_ (appropriate 0.2, 0.6, 1.0, 2.0, and 3.0). At this time, a clear gradient refers to a V-shaped graph, in which the coverage is high near the replication origin and low at the terminus region.

**Fig. 2:**
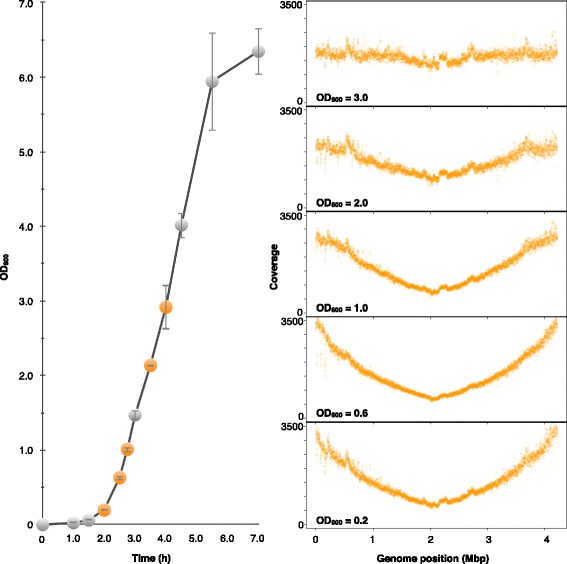
The trend in the DNA copy number gradient at each growth phase. The orange plots on the growth curve are the sequencing points. The right panels are the read coverage obtained by the mapping of the sequenced reads. When the OD_600_ was above 2.0, the coverage gradient disappeared

Until the OD_600_ reached 0.6, the V-shaped coverage gradient was clearly observed (Fig. 2). When the OD_600_ was 1.0, the coverage gradient was not observed throughout the genome. Although the coverage is shown as a V-shaped graph around the replication terminus, the coverage was uniform near the replication origin region. When the growth stage exceeded 3.0, the coverage differences disappeared between the genomic positions. To verify the influence of the coverage gradient on the assembly, we performed the assembly at each time point read. As a result, we confirmed that coverage differences produced by growth timing did not have a big influence on assembly (Additional file 1: Table S1).

### Demonstration of eRP arrangement

**Fig. 3:**
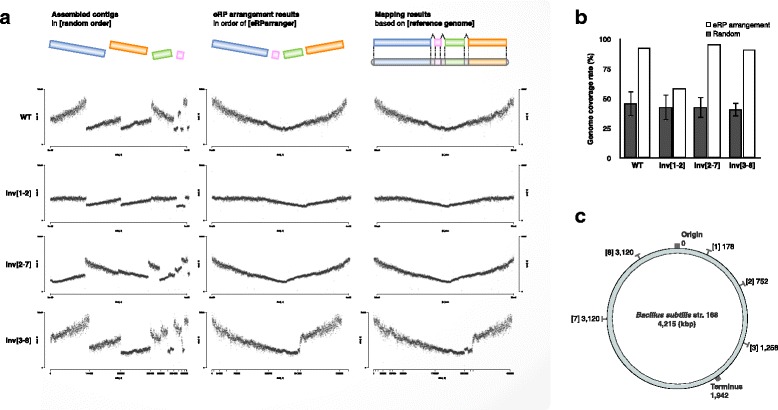
**a** The conceptual rearrangement figures and read coverage graphs on the arranged contigs. From the left panels, in order of contig length, the rearrangement results from the eRP arrangement algorithm and the correct order are shown using the reference genome information as the control. Four strains including one *B. subtilis* str. 168 (WT) and three inversion mutants (Additional file 2: Table S2) are used for the demonstration. **b** The arrangement accuracy in each strain. The genome coverage was calculated by dot plot graph for each order. The genome coverage rates were calculated as a percentage of the genome coverage of the eRP arrangement order to the coverage of the correct order. As the negative control, a randomly shuffled contig order and orientation was used (total of 100 replicates). **c** Construct information about the inverted mutants

To demonstrate the eRP arrangement, we used *B. subtilis* str. 168 (WT) and various mutants (Additional file 2: Table S2). We sequenced and assembled the sampled genomic DNA at the appropriate times according to the above results and obtained just over a dozen contigs. The assembled results are shown in Additional file 3: Table S3. The coverage gradient on each contig was calculated by short-read mapping and is represented in the upper left of Fig. 3a. The contigs in the figure were arranged by length in descending order, and it was obvious that the coverage gradient was uneven. Here, we developed an algorithm to arrange the contigs so that the coverage gradient is consecutive. This algorithm does not require the reference genome, and it sorts the contigs by considering only the coverage continuity. A detailed description is included in the “Methods” section. This algorithm was implemented as an eRP arrangement program. The above contigs that were arranged using the program are shown in the upper middle panel of Fig. 3a. The results show a theoretical V-shaped arrangement, in which the coverage was high at both ends (replication origin) and low in the center (replication terminus). The upper right panel of Fig. 3a shows when the contigs were actually arranged in the correct order, and the orientation was based on the contig mapping onto the genome sequence data. For the quantitative evaluation, we calculated the genome coverage rate with a dot plot graph and compared the rates between the contigs that were rearranged by eRP arrangement and randomly shuffled contigs. As a result, the genome coverage of the arranged contigs by eRP arrangement was greater than 92% correct (Fig. 3b). Furthermore, we demonstrated the utility of eRP arrangement in other species (*E. coli*, *Enterococcus faecalis*, and *Lactobacillus gasseri*). We showed that the eRP arrangement is adaptable and that there were no species-specific limitations if the samples were sequenced with the appropriate timing (Additional file 4: Figure S1 and Additional file 5: Table S4).

### A case study on the effects of the altered genomic structure

The eRP arrangement strategy was applied to the three mutants whose genomic structures were collapsed by inversion mutation. Three mutants were named inv.[n1-n2], where n1 and n2 indicate the endpoints of the inversion regions (Fig. 3c). The graphs in the lower three rows in Fig. 3a are the coverage gradients of the contigs in descending order by length, and then, they were arranged by our method and correctly ordered and oriented. In all cases, the results of the eRP arrangement were better than they were after the randomly shuffled arrangement (Fig. 3b).

## Discussion

The eRP arrangement strategy enable large-scale contig arrangement that allows for an observation of the genomic structure using the replication behavior that is common to all living things without requiring sequence information. The base composition bias, skewed oligomers, and gene directions are representative of biological information that is related to the genomic structure. However, in the case of de novo genome sequencing, there are not many cases in which the gene direction or replication origin and terminus are clearly annotated. This strategy overcomes these limitations by employing replication behavior in the genome assembly.

The use of biological information in genome assembly or scaffolding has become more common since the introduction of GFinisher, a tool that use the base compositional bias called GC skew [34]. In this study, we utilized the intracellular replication behavior as new biological information. Our research revealed that the replication behavior could clearly be observed by eRP if the OD_600_ was less than 1.0 (Fig. 2). This tendency can be stably observed with various bacteria [32]. Accordingly, when we used the extracted genomic DNA at the appropriate growth phase, the strategy had a high accuracy (Fig. 3b). Moreover, its applicability was confirmed using various mutants. In the case of inv. [1, 2], since the growth was slow and no extreme difference was observed in the DNA copy number on the genome, the coverage gradient of the inv. [1, 2] strain was relatively flat. In the case of the inv. [3–8] strain, a pronounced shift point was observed in the coverage gradient graph. The shift point in the v-shape indicates replication fork pausing. This phenomenon has also been discussed in previous studies [28] and is commonly seen in mutants whose genomic symmetry has been disrupted. However, according to the results in Fig. 3b, our method succeeded in rearranging the contig with higher accuracy than a random rearrangement in both strains. Thus, the eRP arrangement approach was applicable to various strains with slow growth or a collapse in genomic polarity. The scope of the eRP arrangement strategy application is not limited to model organisms with fast growth rates. Even if the cell cycle is not synchronized, if genome replication is in progress in most cells, the difference in the DNA copy number between the replication origin and terminus is greater than 1 according to a Cooper-Helmstetter model [35, 36]. Furthermore, this model and the speed of the bacterial replication fork [37] are mentioned in consideration of the relationship between the growth phase and assembly. In general, sequencing during the exponential phase may negatively affect the assembly because the high-coverage region is seen as a repeat and the low-coverage region is an artifact from the k-mer. However, considering the replication speed and Cooper-Helmstetter model, the coverage difference at the exponential phase is expected to be less than three times at maximum. In fact, uneven coverage was not large enough to affect adversely the assembly (Additional file 1: Table S1). Furthermore, this strategy was also validated in mutants (Fig. 3b). The mutants used in the demonstration have approximately the same genome sequences and alter only the genomic structure by inversion mutation.

The advantage of our method is that it only requires sampling with proper timing and simple de novo sequencing, and it does not require additional sequencing. This eRP arrangement approach does not combine special sequencing, and it only requires a conventional library preparation and sequencing at the appropriate sampling point. These advantages will be useful for many projects such as comprehensive de novo genome sequencing of bacteria [38].

However, the eRP arrangement strategy does have several requirements. First, because the gradient of the DNA copy number of short contigs is difficult to calculate, the accuracy of the eRP arrangement is highly dependent on the properties of the contigs in use. Using *B. subtilis* WT, we show the relationship between the number of contigs and accuracy in Additional file 6: Table S5. Next, the experimental condition that affects the accuracy is the sequencing depth. The number of sequence reads used in the demonstration was approximately 20 M reads, and the genome coverage of *B. subtilis* (4.2 Mbp) was 450X. Because low-depth sequencing decreases the accuracy of the assembly and eRP calculation, our system might require high sequencing coverage of approximately 100X or more (Additional file 7: Figure S2). The number of reads also affects the contig number. Because our system does not use a short contig with a length that is less than approximately 1% of its genome size, it cannot correspond to more than approximately 100 contigs. In these cases, reassessment of the assembly tools is required. Additionally, since the current version targets only one independent replicon, it can not be used for plasmids or multiple chromosomes.

This demonstration shows that the eRP arrangement was effective against the inverted mutants, indicating that our strategy is useful for de novo sequencing in closely related species with different genomic structures. De novo genome sequencing is expected to become more popular in microbiology. The reference or related genome-free method will contribute to this field in the future.

## Conclusions

We present a novel eRP arrangement strategy for the analysis of the positional relationships between assembled contigs in bacterial circular chromosomes. The eRP arrangement makes it possible to order and orient the assembled contigs from de novo bacterial genome sequencing using the universal biological features of bacteria for reference. We believe that this strategy will assist in the further acceleration of genome sequencing and the growth of comparative analysis for genomic structures in microbiology. The arrangement algorithm program is freely available at https://github.com/nkono/eRParranger **.**

## Methods

### Strains and antibiotic conditions

All strains were derived from *B. subtilis* str. 168 (hereinafter called WT, Additional file 2: Table S2). The inversion mutants were isolated using a *ne*-*eo* system, which has been described previously [28, 30, 39]. Agar medium was prepared by adding agar (1.5% *w*/*v*) to Luria-Bertani (LB) broth with supplements of 250 μg/ml blasticidin S (BS), 50 μg/ml spectinomycin (Spc), 10 μg/ml tetracycline (Tet), 5 μg/ml chloramphenicol (Cm), or 5 μg/ml neomycin (Nm).

### Culturing condition

Each colony was inoculated in 2 ml of pre-warmed LB with appropriate antibiotics and incubated with shaking at 180 rpm at 37 °C for 16 h. Samples from a pre-cultured strain that were grown overnight (16 h) in LB broth were diluted by 5% in 50 ml of pre-warmed LB broth in a 200-ml flask and incubated at 37 °C until each strain reached exponential phase. The OD_600_ was calculated every 30 or 60 min. The eRP feasibility tests (Fig. 2) were performed when OD_600_ values of approximately 0.2, 0.6, 1.0, 2.0 and 3.0 were reached.

### Library preparation and sequencing

The genomic DNA was purified using phenol-chloroform extraction and ethanol precipitation from each culture at appropriate time points. In the case of the feasibility study regarding the DNA copy number gradient during different growth phases (Fig. 2), the sequence library was prepared using the standard protocol for the KAPA HyperPlus Kit (for Illumina), and sequencing was performed with a NextSeq 500 instrument (Illumina, Inc.) using a 75 bp single-end read. For the eRP demonstration (Fig. 3), the sequence library was prepared using a standard protocol with the Nextera DNA Library Preparation Kit (Illumina, Inc.), and sequencing was performed with a GAIIx instrument (Illumina, Inc.) using a 100 bp paired-end read. All reads were used for each assembly. The quality of the sequencing results was assessed with FastQC (v0.10.1) [40]. The data sets obtained from this study were deposited and are available at the DNA Data Bank of Japan (DDBJ: http://www.ddbj.nig.ac.jp/) Sequence Read Archive with Accession no. DRA005896 (Additional file 8: Table S6).

### eRP arrangement demonstration in other species

For the demonstration in other species, we selected these three species (*E. faecalis*, *L. gasseri*, and *E. coli*), because their genomes had been sequenced at exponential growth phase previously and the number of inexact repeats in the genome was diverse. The sequencing data were obtained from the NCBI Sequence Read Archive (SRA) under accession numbers ERR969340 and ERR969426 [32] for *E. faecalis* and *L. gasseri*, and SRX703252 for *E. coli*.

### Computational analysis

All bioinformatics analyses were conducted using G-language GAE, version 1.9.1 [41]. The visualizations were performed using the R statistics package version 3.2.1. The *B. subtilis* str. 168 (WT) genome (NC_000964.3: 31-DEC-2013) sequence was obtained from the National Center for Biotechnology Information FTP Repository. The assembly was performed using SPAdes v3.7.1 [9, 42]. The reads and contig mapping were performed using BWA 0.7.11-r1034 [43]. The inexact repeat was defined as over 300 bp region and the number of repeats was calculated by a nucmer alignment script [44].

### eRP validation

To validate the eRP arrangement results, first we prepared the correct order and calculated the genome coverage. The correct order used for verification was obtained by contig mapping onto the reference genome sequence of each strain using BWA MEM 0.7.11-r1034 [43]. The genome coverage was calculated by performing a large-scale alignment between the arranged contigs and the reference genome with a dot plot graph. The large-scale alignment was performed by MUMmer 3.23, with a uniqueness of 95 [44]. The genome coverage rate for the eRP validation in Fig. 3b and Additional file 4: Figure S1 was calculated as a percentage of the genome coverage of the eRP arrangement order compared to the coverage of the correct order. A randomly shuffled contig order and orientation was used as the negative control (total of 100 replicates).

### eRP arrangement overview and algorithm

The eRP arrangement is a new strategy for understanding the genomic structure in bacteria by arranging the contig order and orientation. The eRP arrangement strategy outlines procedures for genomic DNA extraction and contig rearrangement. An overview of the eRP arrangement can be described as follows: (1) Extract the genomic DNA at an appropriate growth phase for clear gradient coverage throughout the entire genome, (2) prepare the library and sequences using a massively parallel sequencer and assemble the short reads, (3) map the reads onto the assembled contigs and calculate the coverage, and (4) arrange the order and orientation of the contigs in a continuous coverage gradient (Fig. 1). The detailed algorithm in (3–4) is as follows: first, we calculated the slopes of the linear regression lines from the read coverage in contigs using the least squares method. Step 1: align the negative contig slope and the contig with the highest coverage as the base contig. Step 2: select the contig with the next smallest coverage difference from the base contig and connect it with the base contig. Step 3: when there are no next contig candidates, then the assembly step has reached a shift point in the V-shaped coverage graph. Then, rotate the remaining contigs and repeat step 2 while looking for the next smallest positive coverage difference. This source code is freely available at https://github.com/nkono/eRParranger.

## Additional files

Additional file 1: Table S1. Assemble data in each growth phase (OD_600_). Related to Fig. 2. (XLSX 27 kb)

Additional file 2: Table S2. All strain information. (XLSX 22 kb)

Additional file 3:Table S3. Assemble results in each *B. subtilis* strain. Related to Fig. 3. (XLSX 29 kb)

Additional file 4: Figure S1. eRP demonstration in other species (*Escherichia coli*, *Enterococcus faecalis*, and *Lactobacillus gasseri*). These raw sequence data were obtained from the NCBI Sequence Read Archive (SRA) under accession numbers ERR969340 and ERR969426 for *E. faecalis* and *L. gasseri* and SRX703252 for *E. coli*. The left panels provide the mean sequence coverage on each contig, in the order of the contig length and rearrangement results from the eRP arrangement algorithm. The right graph is the arrangement accuracy in each result. The error bars indicate the mean SD. (PDF 658 kb)

Additional file 5: Table S4. eRP arrangement result data in other bacteria. Related to Figure S1. (XLSX 31 kb)

Additional file 6: Table S5. eRP arrangement accuracy corresponding to the number of contigs. (XLSX 24 kb)

Additional file 7: Figure S2. The relationship between the genome coverage rate and the number of reads used for eRP arrangement. The arrangement quality declined depending on the number of read. (PDF 22 kb)

Additional file 8: Table S6. DDBJ Sequence read archive attributes. (XLSX 29 kb)

## Abbreviations

eRP: experimental replication profiling
