# Supplementary figures and images for: eRP arrangement: a strategy for assembled genomic contig rearrangement based on replication profiling in bacteria
Source: BMC Genomics. 2017 Oct 13;18:784. doi: 10.1186/s12864-017-4162-z (PMC5640929; doi:10.1186/s12864-017-4162-z)

**Assembled contigs  
in [random order]**

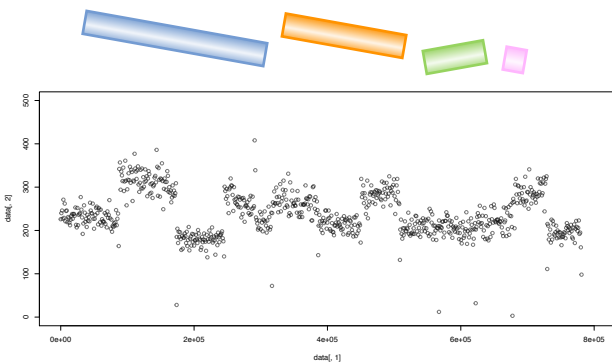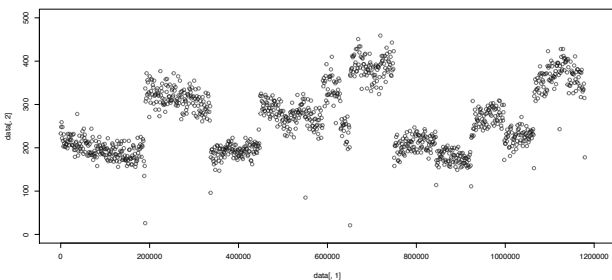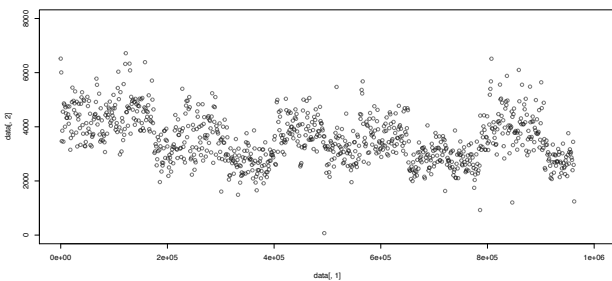

**eRP arrangement results  
in order of [eRParranger]**

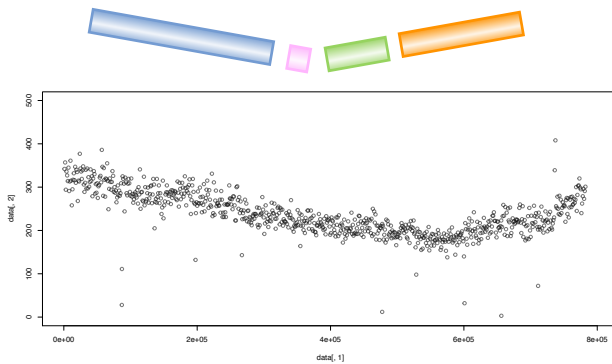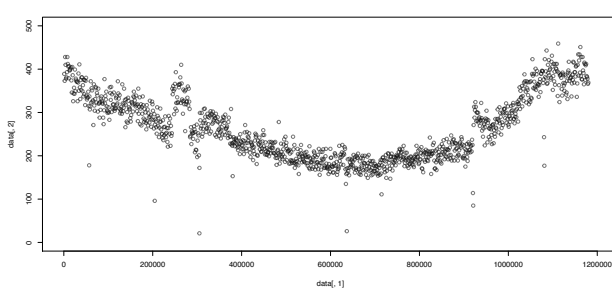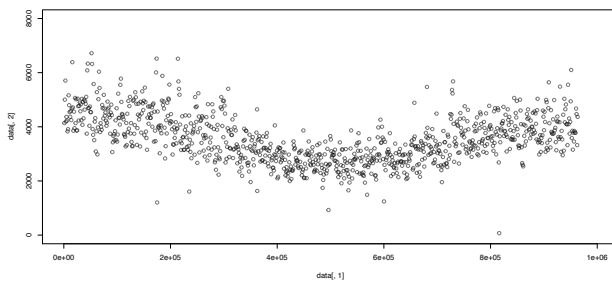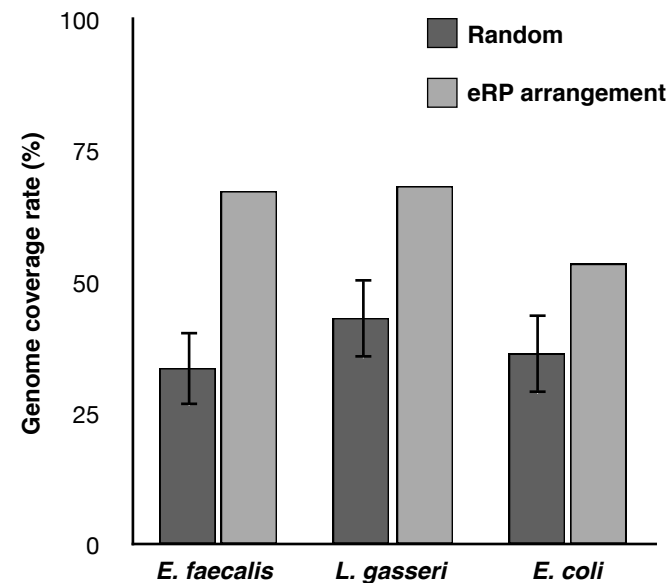

Supplement: Supplementary file 4 — eRP demonstration in other species (Escherichia coli, Enterococcus faecalis, and Lactobacillus gasseri). These raw sequence data were obtained from the NCBI Sequence Read Archive (SRA) under accession numbers ERR969340 and ERR969426 for E. faecalis and L. gasseri and SRX703252 for E. coli. The left panels provide the mean sequence coverage on each contig, in the order of the contig length and rearrangement results from the eRP arrangement algorithm. The right graph is the arrangement accuracy in each result. The error bars indicate the mean SD. (PDF 658 kb) [file 12864_2017_4162_MOESM4_ESM.pdf]

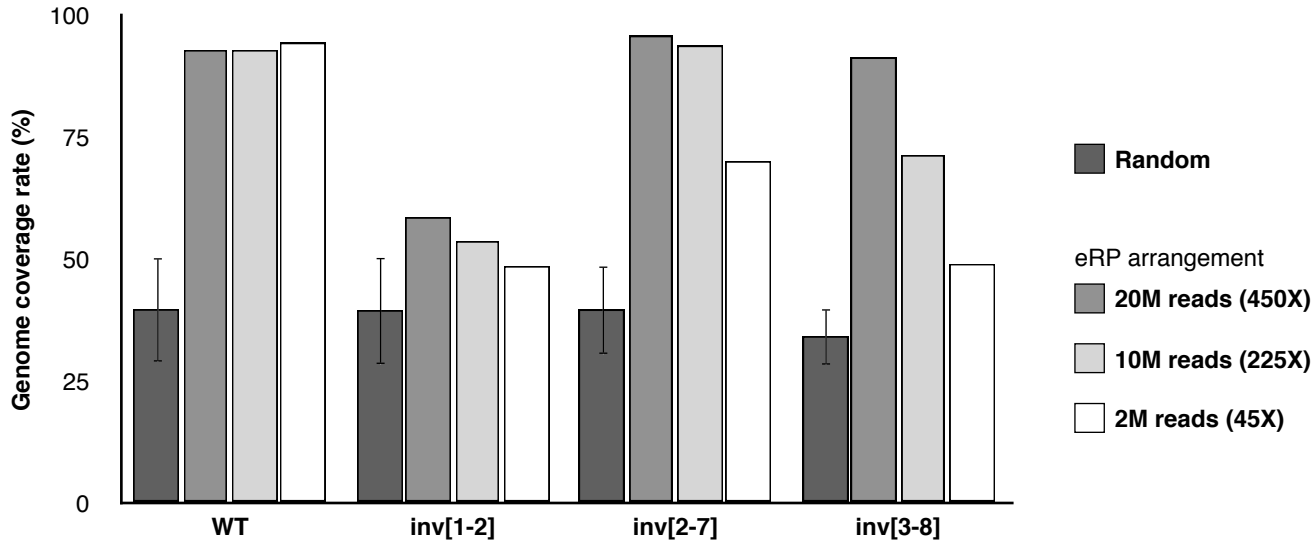

Supplement: Supplementary file 7 — The relationship between the genome coverage rate and the number of reads used for eRP arrangement. The arrangement quality declined depending on the number of read. (PDF 22 kb) [file 12864_2017_4162_MOESM7_ESM.pdf]
